# Artificial intelligence in transarterial chemoembolization for hepatocellular carcinoma: from treatment response prediction to refractoriness assessment

**DOI:** 10.3389/fonc.2026.1822689

**Published:** 2026-05-29

**Authors:** Xiao Ma, Jiaping Wang, Heng Li

**Affiliations:** 1 Department of Thoracic Surgery II, The Third Affiliated Hospital of Kunming Medical University, Kunming, Yunnan, China; 2 Department of Interventional Radiology, The Second Affiliated Hospital of Kunming Medical University, Kunming, Yunnan, China

**Keywords:** artificial intelligence, deep learning, hepatocellular carcinoma, prognostic assessment, radiomics, TACE refractoriness, transarterial chemoembolization, treatment response prediction

## Abstract

**Background:**

Hepatocellular carcinoma (HCC) is the most prevalent primary hepatic malignancy worldwide and a leading cause of cancer-related mortality. Transarterial chemoembolization (TACE) is the standard of care for Barcelona Clinic Liver Cancer (BCLC) intermediate-stage HCC; however, treatment response varies considerably among individuals, and a substantial proportion of patients develop TACE refractoriness.

**Objective:**

This review systematically examines the current applications of artificial intelligence (AI) in TACE for HCC, encompassing treatment response prediction, survival prognostication, refractoriness prediction, and the underlying molecular mechanisms, while critically appraising methodological quality of the existing literature and delineating TACE-specific future directions.

**Methods:**

We conducted a comprehensive literature search of PubMed, Embase, Web of Science, Scopus, and Google Scholar for studies published through 2025 that investigated AI, machine learning (ML), or deep learning (DL) in TACE response prediction, prognostic stratification, and refractoriness assessment. Included studies were appraised against PROBAST, IBSI, TRIPOD+AI, and CLAIM frameworks.

**Results:**

AI models based on radiomics and DL demonstrated high discriminative performance for predicting TACE outcomes, with meta-analytic area under the receiver operating characteristic curve (AUROC) values ranging from 0.81 to 0.92. Combined clinico-radiological models—incorporating albumin–bilirubin (ALBI) grade, BCLC stage, alpha-fetoprotein (AFP) level, tumor diameter, distribution, and peritumoral arterial-phase enhancement—consistently outperformed single-source models. Convolutional neural networks (CNNs) and gradient-boosting, support-vector-machine, and random-forest models were the consistently top-performing algorithms. However, methodological quality was uneven: most studies showed high or unclear PROBAST risk of bias in the analysis domain, IBSI-compliant feature reporting and public code release were rare, and the generalization gap between internal and external validation cohorts averaged 0.08–0.15 AUROC.

**Conclusions:**

AI offers a powerful toolkit for individualized TACE decision-making, with the potential to shift clinical practice from experience-driven to data-driven precision therapy. Nevertheless, critical challenges remain—including insufficient external validation, limited sample sizes, geographic bias, low standardization, and inadequate interpretability—which together necessitate large-scale, multicenter, prospective studies adhering to harmonized reporting standards.

## 1 Introduction

Primary liver cancer constitutes a major global public health burden, ranking among the leading causes of cancer-related mortality. HCC accounts for 75–85% of all primary liver cancers and is particularly prevalent in East Asia, where chronic hepatitis B virus (HBV) infection is the predominant etiological factor (1, 2). Under the BCLC staging system, TACE is the recommended first-line treatment for patients with intermediate-stage (BCLC-B) HCC (3, 4). In addition, TACE plays an important role in selected early-stage patients who are ineligible for surgical resection and in appropriately selected advanced-stage patients (5).

TACE exerts a dual antitumor effect by selectively delivering a mixture of chemotherapeutic agents and embolic materials via the hepatic artery: direct cytotoxic damage from the chemotherapeutic agent is coupled with ischemic necrosis resulting from arterial occlusion (6). Conventional TACE (cTACE) employs an emulsion of lipiodol and chemotherapeutic agents together with gelatin sponge particles, whereas drug-eluting bead TACE (DEB-TACE) uses drug-loaded microspheres to achieve sustained, controlled drug release (7). The overall objective response rate of TACE ranges from 40% to 60%, with marked interindividual heterogeneity (8, 9).

A subset of patients exhibit persistent tumor progression or an inadequate response despite multiple TACE sessions—a clinical scenario termed “TACE refractoriness.” The Japan Society of Hepatology (JSH) first proposed formal criteria for TACE refractoriness in 2010, subsequently revised in 2014 and 2021 (10–12). The 2022 BCLC strategy update recommends timely transition to systemic therapy—including tyrosine kinase inhibitors or immune-checkpoint inhibitors—for patients who become refractory to TACE (13). However, the definition of TACE refractoriness remains a subject of debate across different regions and clinical guidelines (14, 15).

**Figure 1:**
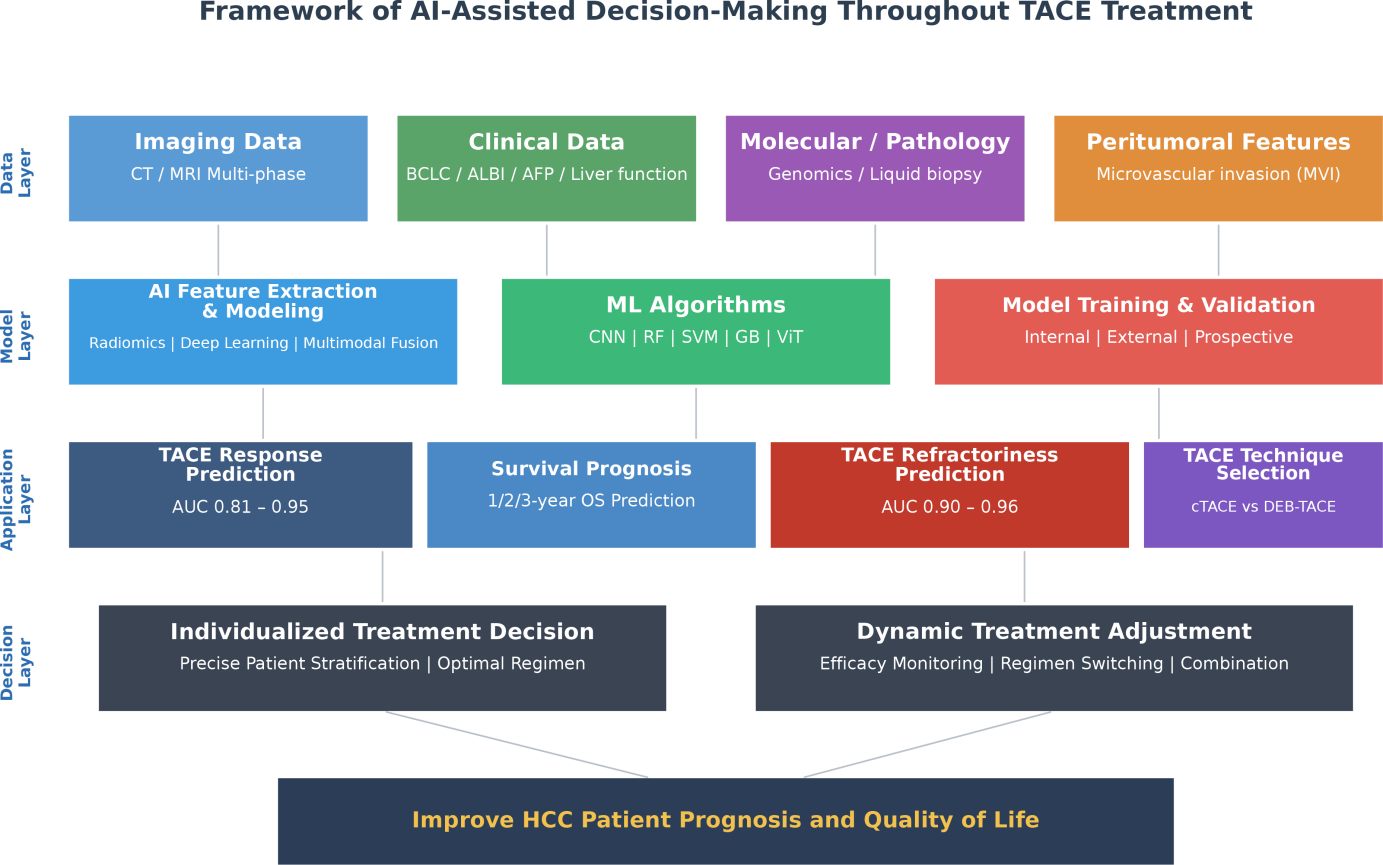
Framework of AI-assisted decision-making throughout the TACE treatment continuum. The workflow spans multisource data acquisition prior to TACE, AI-driven feature extraction and model development, multi-scenario clinical applications, and individualized therapeutic decision-making.

Recent advances in AI, particularly in ML and DL, have opened new avenues for medical image analysis and clinical decision support. Radiomics enables high-throughput extraction of quantitative features from medical images, capturing tumor heterogeneity information imperceptible to the human eye (16, 17). DL methods—especially CNNs—can automatically learn hierarchical feature representations directly from raw imaging data, bypassing the need for manual feature engineering (18, 19). These AI techniques offer promising tools for precise TACE outcome prediction and individualized treatment planning. The present review aims to provide a comprehensive overview of AI applications across the entire TACE treatment continuum for HCC, from pretreatment response prediction to refractoriness assessment (**Figure 1**), to critically appraise the methodological quality of the underlying evidence base, and to delineate TACE-specific future directions.

## 2 Overview of artificial intelligence techniques

### 2.1 Machine learning and deep learning

ML, a core branch of AI, seeks to enable computational systems to learn and improve from data without explicit programming. Supervised learning algorithms most commonly employed in TACE outcome prediction include logistic regression (LR), support vector machines (SVMs), random forests (RFs), gradient boosting (GB), and K-nearest neighbors (KNN) (20, 21). These algorithms can model complex, nonlinear relationships between high-dimensional input features and clinical endpoints.

DL, a subfield of ML, leverages deep artificial neural networks to automatically learn hierarchical feature representations from raw data. CNNs remain the most widely adopted DL architecture in medical image analysis, while attention-based models such as Vision Transformers (ViTs) have recently gained traction (22, 23). A key advantage of DL is its ability to bypass conventional handcrafted feature extraction and selection, directly learning outcome-relevant representations from imaging data (24).

### 2.2 Radiomics methodology

Radiomics is a high-throughput computational approach that converts medical images (CT, MRI) into mineable quantitative data by extracting a large number of features, including morphological descriptors, first-order statistics, texture features, and wavelet-transformed features (16, 25). The standard radiomics pipeline comprises image acquisition and preprocessing, region-of-interest (ROI) segmentation, feature extraction, feature selection and dimensionality reduction, and predictive model construction with validation (26). The least absolute shrinkage and selection operator (LASSO) regression is the most frequently used feature selection method in TACE outcome prediction, adopted by approximately 60.8% of published studies (27). The Image Biomarker Standardization Initiative (IBSI) aims to harmonize feature extraction across different software platforms to ensure reproducibility and comparability; however, many existing studies have not rigorously adhered to IBSI standards, thereby limiting cross-study result transferability (28, 29).

### 2.3 Methodological quality of the included evidence

To contextualize the performance claims summarized in subsequent sections, we appraised the included primary studies against four established frameworks: the Prediction model Risk Of Bias ASsessment Tool (PROBAST) for prediction-model studies (30); IBSI compliance for radiomic feature extraction and reporting (29); the TRIPOD+AI 2024 statement, which supersedes the 2015 TRIPOD checklist and provides harmonized reporting guidance for both regression-based and machine-learning prediction models (31); and the Checklist for Artificial Intelligence in Medical Imaging (CLAIM), which addresses imaging-specific reproducibility elements (32). The complementary QUADAS-AI tool for AI-centered diagnostic test accuracy studies, currently in consensus development, will further strengthen this appraisal landscape once finalized (33).

Across the studies summarized in **Table 1**, the majority demonstrated high or unclear PROBAST risk of bias in the analysis domain, most commonly due to (a) sample sizes below recommended events-per-variable thresholds, (b) absence of independent external validation, and (c) lack of pre-specified analysis plans. IBSI-compliant reporting of feature definitions was explicit in only a minority of studies (27, 28). Code availability was rare: fewer than 15% of cited studies released their training code or trained model weights in a public repository. ROI segmentation was predominantly manual or semi-automatic using proprietary software, with ITK-SNAP and 3D Slicer being the most common open-source exceptions; fully automatic, open-source segmentation pipelines (e.g., nnU-Net–based) were used in only a handful of recent studies. These limitations collectively constrain reproducibility and cross-study comparability, and they should be addressed in future work through pre-registration, public code release, and adherence to TRIPOD+AI and CLAIM reporting standards.

**Table 1:** Summary of representative studies on AI models for predicting TACE outcomes, with methodological quality indicators.

Study	n	Imaging	Algorithm	Endpoint	AUC (Train)	AUC (Val)	Validation	IBSI	Code	PROBAST RoB
Chen 2024 (34)	173	CT	LASSO + Nomogram	Early recurrence post-PA-TACE	0.853	0.929	Internal	Unclear	No	High (analysis)
Zhao 2025 (36)	122	CT	LR	First TACE efficacy	0.92	0.815	Internal	Unclear	No	High (analysis)
Wang J 2025 (39)	n/r	MRI	DL + handcrafted radiomics	Peritumoral recurrence post-DEB-TACE	0.802	0.770 (external)	External	Unclear	No	Unclear
Yao 2025 (40)	181	MRI	ProgSwin-UNETR (Transformer)	Four-class prognosis stratification	0.92	C-index 0.81	Internal	N/A (DL)	No	Unclear
Liu 2025 (41)	475	CT	Random forest + DL	Distant metastasis risk	0.931	0.854 (external)	External	Unclear	No	Low–Unclear
Peng 2020 (43)	789	CT	ResNet50 (transfer learning)	Objective TACE response	0.95–0.97	0.95–0.97 (multi-center)	External (2 centers)	N/A (DL)	No	Unclear
Dong 2025 (45)	166	MRI	LR	TACE refractoriness	0.955	0.941	Internal	Unclear	No	High (sample size)
Li H 2025 (46)	108	CT	SVM	Post-refractory prognosis	0.956	0.897	Internal	Unclear	No	High (sample size)
Kiani 2025 meta (42)	27 studies	CT/MRI	Multiple	TACE response	0.89 (pooled)	0.81 (pooled)	Mixed	Mixed	Mixed	Mixed

LR, logistic regression; RF, random forest; DL, deep learning; SVM, support vector machine; LASSO, least absolute shrinkage and selection operator; n/r, not reported. PROBAST RoB judgments reflect appraisal of the analysis domain in particular; values were derived from study text. “IBSI” denotes explicit, verifiable IBSI-compliant reporting; “unclear” indicates that the use of an IBSI-validated extractor (e.g., PyRadiomics) was stated but specific feature compliance was not documented.

## 3 AI in TACE treatment response prediction

### 3.1 Radiomics-based response prediction

A substantial body of evidence supports the use of radiomics for predicting TACE response in HCC. A systematic review encompassing 23 studies reported that radiomics-based AI models (67 methods) achieved a median AUC of 0.79 (95% CI: 0.75–0.82), significantly outperforming non-radiomics models at 0.73 (95% CI: 0.61–0.77) (27).

In CT-based radiomics, several studies have evaluated the impact of different contrast-enhancement phases and regions of interest on predictive performance. Chen et al. developed a multimodal dual-region CT radiomics model integrating features from both the primary tumor and the spleen to predict early recurrence (ER) after postoperative adjuvant TACE (PA-TACE) in 173 patients, achieving AUCs of 0.853 (95% CI: 0.783–0.908) in the training set and 0.929 (95% CI: 0.789–0.988) in the validation set; multivariate analysis identified total bilirubin and the combined Rad-score as independent predictors (34). Guo et al. constructed a multi-parametric MRI-based radiomics model for predicting recurrence after PA-TACE, with AUC values of 0.82–0.91 for 1-year recurrence prediction; the combined model integrating Rad-score, NLR, and tumor size outperformed clinicopathologic models alone (35). Zhao et al. evaluated intratumoral and peritumoral CT radiomics for predicting first TACE efficacy in 122 patients with advanced HCC, reporting that the combined clinical-radiomics model achieved AUCs of 0.92 (95% CI: 0.87–0.95) in the training cohort and 0.815 (95% CI: 0.67–0.95) in the validation cohort, significantly outperforming the clinical model alone (36).

In MRI-based radiomics, quantitative MRI (qMRI) provides multidimensional information for TACE outcome evaluation. Diffusion-weighted imaging (DWI), diffusion kurtosis imaging (DKI), intravoxel incoherent motion (IVIM) imaging, and perfusion imaging have all been applied to predict and assess TACE efficacy (37). Reported discriminative performance varies widely across qMRI modalities, and isolated single-center studies have claimed near-perfect discrimination (e.g., AUC approaching unity for combined magnetic resonance spectroscopy and DWI in early-response assessment); such results, however, almost invariably reflect very small validation cohorts, feature selection performed without held-out testing, or other forms of optimistic bias, and should be regarded as hypothesis-generating rather than as evidence of clinical readiness. Wen et al. developed a multiparametric MRI-based nomogram integrating intra- and peritumoral radiomic signatures with four clinical predictors (HBsAg, AFP, BCLC staging, and tumor size) to predict complete response to initial TACE in virus-associated HCC, yielding AUCs of 0.892, 0.851, and 0.787 in the training, internal-validation, and external-validation cohorts, respectively (38).

### 3.2 Deep-learning–based response prediction

DL approaches offer complementary strengths to conventional radiomics for TACE outcome prediction. Wang et al. combined DL radiomics with handcrafted radiomics utilizing contrast-enhanced MRI to predict early peritumoral recurrence after DEB-TACE, with the best model achieving AUCs of 0.802 (95% CI: 0.718–0.887) in the training cohort and 0.770 (95% CI: 0.623–0.916) in the external-validation cohort (39). Yao et al. developed the ProgSwin-UNETR model—a transformer-based architecture for four-class prognosis stratification from multi-time-point arterial-phase MRI in 181 HCC patients (543 scans)—achieving an accuracy of 0.86 and an AUC of 0.92 (95% CI: 0.90–0.95), with a *post hoc* Cox regression C-index of 0.81 (40). Liu et al. developed a combined clinical-radiomics-DL (CRDL) model for predicting distant metastasis risk in 475 HCC patients after TACE, with AUC values of 0.931, 0.861, and 0.854 in the training, testing, and external-validation cohorts, respectively (41).

A systematic review and meta-analysis by Kiani et al., incorporating 27 studies, evaluated the predictive performance of AI models for TACE treatment response. The overall meta-analysis of 11 studies yielded a pooled AUROC of 0.89 (95% CI: 0.81–0.93) for internal validation and 0.81 (95% CI: 0.80–0.92) for external validation, with no significant differences between datasets (P = 0.66) or between DL and handcrafted radiomics models (P = 0.21) (42). In an earlier multicenter study, Peng et al. employed a transfer-learning ResNet50 architecture to predict TACE response from CT imaging in 789 HCC patients across three institutions, reporting per-class AUCs of 0.95, 0.96, and 0.97 for objective response prediction in the training and two external-validation cohorts (43); these values, while high, are derived from patch-level rather than patient-level evaluation and remain at the upper end of plausible discriminative performance.

Recent innovations include habitat analysis combined with ViTs, which leverages multiphasic contrast-enhanced MRI to delineate intratumoral subregions and predict response to TACE plus systemic therapy (44). This approach exploits the spatial distribution of intratumoral heterogeneity, offering a novel avenue for overcoming traditional radiomics bottlenecks.

### 3.3 Combined clinico-radiomic models

Extensive evidence demonstrates that integrated clinico-radiomic models outperform single-source models for TACE response prediction. In a systematic review of 23 studies, CT-based combined models achieved an AUC of 0.79 (95% CI: 0.73–0.89, P = 0.04) and MRI-based combined models achieved 0.81 (95% CI: 0.75–0.88, P = 0.017), both significantly exceeding clinical-only models at 0.60 (95% CI: 0.55–0.75) (27).

**Figure 2:**
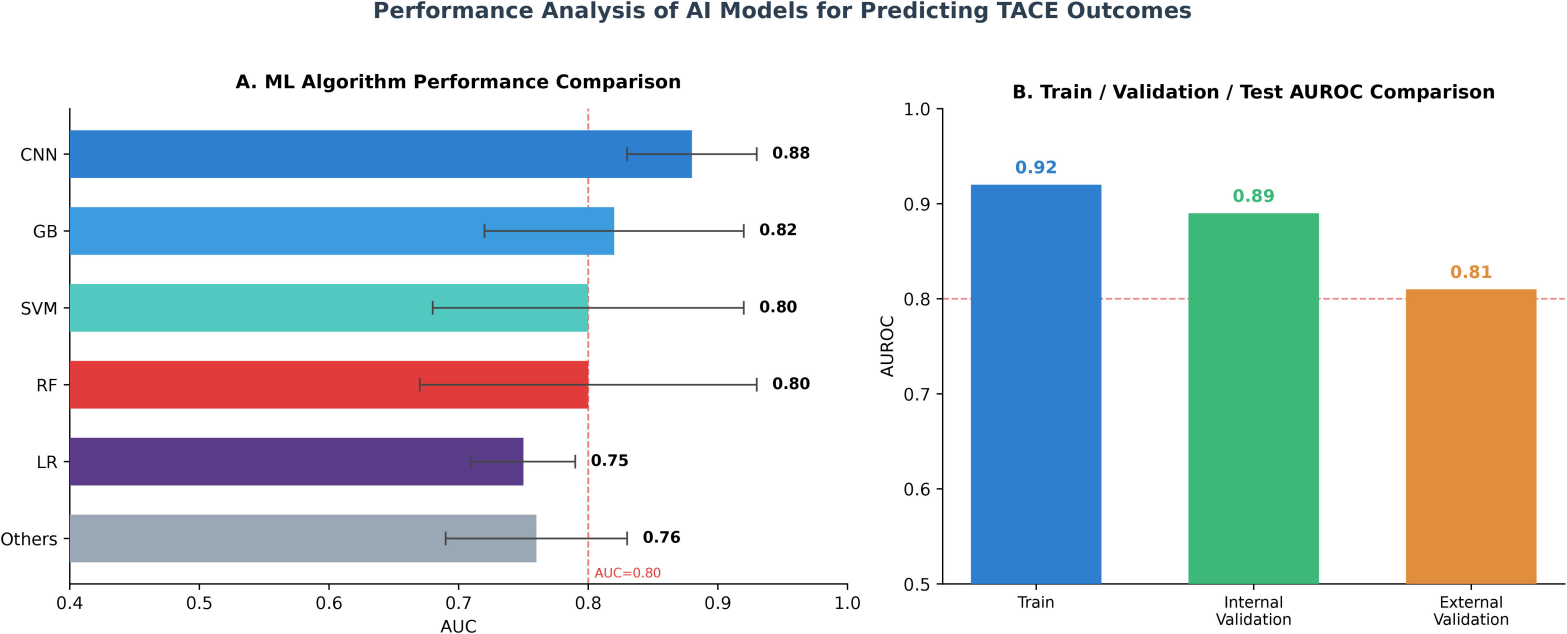
Performance analysis of AI models for predicting TACE outcomes. **(A)** Comparison of AUC values across machine-learning algorithms with 95% confidence intervals. **(B)** Summary AUROC in training, internal-validation, and external-validation datasets, illustrating the generalization gap discussed in §7.1.

The most informative predictors identified through multivariable logistic regression include imaging features—maximal tumor diameter (9 studies), tumor distribution (5 studies), and peritumoral arterial-phase enhancement (4 studies)—and clinical features—ALBI grade (7 studies), BCLC stage (6 studies), and AFP level (6 studies) (27). Augmenting radiomic features with clinical and imaging variables yielded significant performance gains for both CT models (AUC: 0.89) and MRI models (AUC: 0.82) (27). A comparison of discriminative performance across machine-learning algorithms and across training, internal-validation, and external-validation datasets is summarized in **Figure 2**.

## 4 AI in post-TACE survival prediction

Beyond short-term response assessment, AI has demonstrated substantial value in predicting medium- to long-term survival following TACE. Multiple studies have integrated radiomic and/or DL features with clinical parameters using Cox proportional hazards models and Kaplan–Meier analysis to construct 1-, 2-, and 3-year overall survival (OS) prediction models (27, 47).

A retrospective study of 150 patients with advanced HCC receiving TACE combined with camrelizumab and apatinib constructed a CT-based clinical-radiomics nomogram for estimating OS. The combined nomogram demonstrated superior performance with a concordance index (C-index) of 0.840, compared with the radiomics-only nomogram (C-index: 0.817) and the clinical-only nomogram (C-index: 0.661), and exhibited high 1-year and 2-year AUCs of 0.936, confirming that the integration of radiomic and clinical features significantly improves survival prediction for TACE combination-therapy patients (48).

In the context of repeat TACE (re-TACE) decision-making, a novel multidimensional prediction model—the HRD score—has been proposed, integrating radiomic features, DL-derived prediction probabilities, and clinical variables (e.g., HBsAg status) to identify patients unlikely to benefit from further TACE and facilitate timely treatment transition (49). Although the model performed well in training and internal-validation sets, AUC declined in external testing, underscoring the need for further optimization of model generalizability.

## 5 TACE refractoriness: definition, impact, and AI prediction

### 5.1 Definitions and controversies

TACE refractoriness refers to a clinical state in which HCC fails to respond meaningfully—or progresses—despite repeated TACE sessions. The JSH 2021 revised criteria define TACE refractoriness as: (a) >50% viable target lesion or new lesion development after ≥2 TACE sessions; (b) new-onset vascular invasion or extrahepatic metastasis; or (c) persistent hepatic function deterioration to Child–Pugh class C following TACE (11, 12). The BCLC 2022 update recommends transitioning refractory patients to systemic therapy (13).

However, the definition of TACE refractoriness varies across regions and guidelines (**Table 2**). In 2022, the Clinical Guidelines Committee of the Chinese College of Interventionalists (CCI) issued an expert consensus advancing a more restrictive, locally-focused definition: TACE refractoriness is recognized only after ≥3 consecutive sessions of standardized, refined TACE in which the intrahepatic target lesion remains in progressive disease (PD) by mRECIST relative to the pre–first-TACE baseline (50). Critically, the CCI consensus stipulates that new intrahepatic lesions, new vascular invasion, or new extrahepatic metastasis emerging after TACE should not, in isolation, constitute refractoriness, on the grounds that these events reflect the natural systemic course of HCC rather than local treatment failure and that ongoing TACE may still benefit patients with controllable intrahepatic disease (50). A 2020–2021 survey of 257 senior Chinese interventional radiologists found that 91.4% rejected the existing JSH-LCSGJ 2014 definition as ill-suited to Chinese HCC populations, and approximately 46.1% continued TACE-based therapy even after identifying refractoriness by the older criteria (15, 50). This persistent clinical uncertainty underscores the urgent need for objective, AI-driven identification tools.

**Table 2:** International comparison of TACE refractoriness definitions.

Guideline	Core definition of TACE refractoriness	Year	Scope
JSH 2010	Viable lesions >50% after two TACE sessions; new intrahepatic lesions; vascular invasion or extrahepatic spread	2010	Japan/Asia
JSH 2014 revision	Above criteria plus deterioration of liver function to Child–Pugh class C; sustained elevation of tumor markers	2014	Japan/Asia
JSH 2021 revision	≥2 TACE sessions with >50% viable target lesions or new lesions; new MVI or extrahepatic metastasis; persistent hepatic deterioration to CP-C	2021	Japan/Asia
BCLC 2022	Transition to systemic therapy (TKIs and/or immunotherapy) upon TACE unsuitability or failure	2022	Europe/International
CCI Chinese consensus	≥3 standardized, refined TACE sessions with intrahepatic target lesion in PD; new intrahepatic lesions/vascular invasion/extrahepatic metastasis are NOT in themselves refractoriness	2022	China
Yang et al. proposal	>50% viable lesions after two TACE sessions alone should not constitute refractoriness (no OS difference: 35 *vs*. 31 months)	2022	Research proposal

### 5.2 Impact of TACE refractoriness on patient survival

Yang et al. conducted a real-world study of 323 treatment-naive HCC patients, of whom 51.1% were diagnosed with early TACE refractoriness after two consecutive TACE sessions. Following propensity score matching (PSM), the median OS in the refractory group was significantly shorter than in the non-refractory group (21 months *vs*. 34 months, P = 0.002) (51). The incidence of early TACE refractoriness by BCLC stage was 36.5% for stage A, 52.9% for stage B, and 75.5% for stage C. Notably, patients with >50% viable lesions after two consecutive TACE sessions did not demonstrate a statistically significant OS difference compared with the non-refractory group (35 months *vs*. 31 months, P = 0.611) (51). These patients typically harbored multiple lesions (63.16%) and larger tumors (mean diameter: 8.14 ± 4.07 cm), suggesting that complete necrosis of all lesions is difficult to achieve even after two technically successful procedures. This finding challenges the JSH criterion of “>50% viable lesions after two consecutive TACE sessions” as a standalone indicator of refractoriness and calls for re-evaluation of current definitional thresholds.

### 5.3 Molecular mechanisms of TACE refractoriness

**Figure 3:**
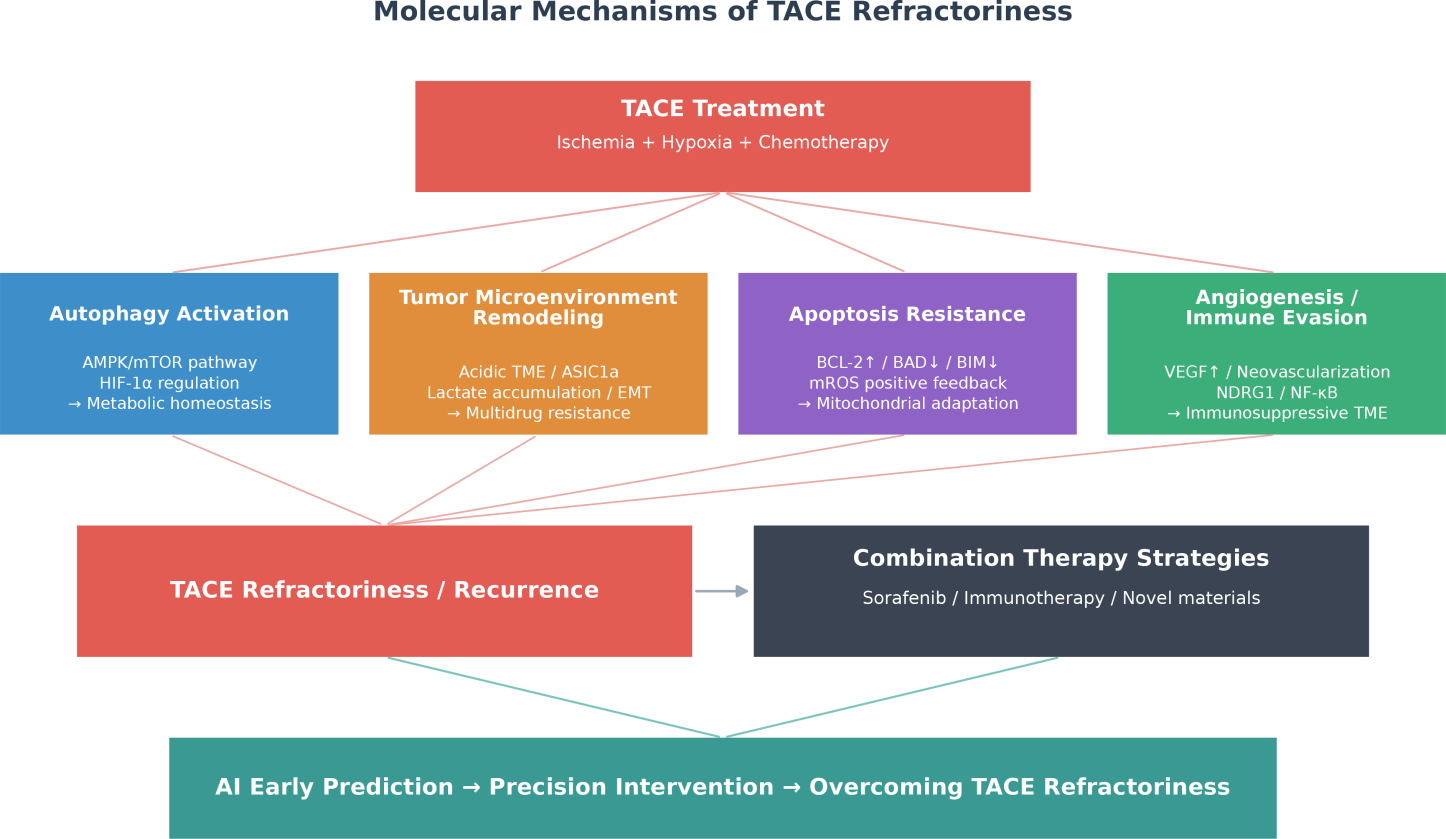
Schematic illustration of the molecular mechanisms of TACE refractoriness and their AI-decodable imaging correlates. The ischemic–hypoxic environment induced by TACE promotes HCC cell survival and tumor recurrence through four principal mechanisms—autophagy activation, tumor microenvironment remodeling, apoptosis resistance, and angiogenesis/immune evasion—each of which produces measurable imaging phenotypes amenable to radiomic and deep-learning analysis, as detailed in **Table 3**.

TACE refractoriness involves complex molecular mechanisms (**Figure 3**). The extreme ischemic–hypoxic conditions induced by TACE activate multiple survival pathways in HCC cells (52):

1. Autophagy-mediated cell survival. TACE-induced ischemia–hypoxia activates autophagy via the AMPK/mTOR pathway and HIF-1α signaling, sustaining cellular energy homeostasis and mediating chemoresistance through metabolic reprogramming (52).
2. Tumor microenvironment remodeling. The acidic post-TACE microenvironment promotes epithelial–mesenchymal transition (EMT) and chemoresistance, with lactate accumulation driving disease progression; the reversed intra–extracellular pH gradient impairs the penetration of weakly basic antineoplastic agents (52).
3. Mitochondrial adaptation and apoptosis resistance. Under hypoxia, HIF-1α upregulates anti-apoptotic BCL-2/BCL-xL while suppressing pro-apoptotic BAD/BIM, conferring apoptotic resistance and supporting mitochondrial adaptation (52).
4. Hypoxia-induced angiogenesis and immune evasion. Post-TACE hypoxia activates vascular endothelial growth factor (VEGF) and other pro-angiogenic signals, driving neovascularization—a major mechanism of post-TACE recurrence—and shaping an immunosuppressive microenvironment (52).

#### 5.3.1 From molecular mechanism to AI-decodable imaging phenotype

Although the molecular drivers of TACE refractoriness described above are not directly observable on routine imaging, each mechanism produces measurable downstream phenotypes that radiomic and DL models can capture, providing the biological rationale for why these models work and how they could be improved. Hypoxia-driven angiogenesis manifests as peritumoral arterial-phase enhancement and corona-enhancement patterns, which are among the most consistently selected features in TACE response models (27). Microenvironmental acidosis and EMT are associated with irregular tumor margins and heterogeneous intratumoral signal, quantifiable through gray-level co-occurrence matrix (GLCM) contrast and entropy as well as neighborhood gray-tone difference matrix (NGTDM) coarseness features. Apoptosis resistance and proliferative aggressiveness correlate with diffusion restriction on DWI (lower apparent diffusion coefficient) and elevated mean kurtosis on DKI. Autophagy and metabolic reprogramming, producing heterogeneous viability across tumor subregions, are amenable to habitat (intratumoral subregion) analysis on multiphasic contrast-enhanced MRI (44). **Table 3** summarizes these mechanism-to-imaging correspondences.

**Table 3:** Mapping molecular mechanisms of TACE refractoriness to AI-decodable imaging phenotypes.

Molecular mechanism	Downstream phenotype	AI-decodable imaging feature	Reported in TACE models
Hypoxia-induced angiogenesis (VEGF↑, HIF-1α)	Neovascularization; arterial-phase hyper-enhancement	Peritumoral arterial-phase enhancement; corona sign; wash-in slope	(27, 36, 38)
EMT and microenvironment remodeling	Irregular margins; infiltrative growth	Shape sphericity; GLCM contrast/entropy; margin irregularity descriptors	(34, 39)
Apoptosis resistance and proliferation	High cellularity; restricted diffusion	ADC↓; DKI mean kurtosis↑; IVIM D↓	(37, 55)
Autophagy and metabolic reprogramming	Heterogeneous viability; intratumoral subregions	Habitat analysis; NGTDM coarseness; wavelet texture	(44)

These correspondences anchor the emerging field of HCC radiogenomics, in which AI models trained on imaging features are used to infer molecular phenotypes such as HIF-1α activity, VEGF expression, immune microenvironment subtype, and oncogenic-pathway activation (53). Incorporating mechanism-informed features—rather than treating radiomics as a purely data-driven black box—is a promising direction for next-generation TACE refractoriness prediction, particularly when paired with liquid biopsy or molecular profiling data for cross-modality validation.

### 5.4 AI in predicting TACE refractoriness

AI offers novel tools for the early prediction of TACE refractoriness. In a multicenter study, Dong et al. extracted radiomic and clinicoradiological features from four MRI sequences, constructing 36 radiomic models; the LR model combining clinical features with multi-sequence radiomics achieved the best performance, with AUCs of 0.955 and 0.941 in the training and validation cohorts, respectively (45). Lu et al. developed an MRI radiomics-based predictor for early treatment response to TACE combined with lenvatinib plus a PD-1 inhibitor in HCC patients with portal vein tumor thrombus (PVTT), with the combined model achieving AUCs of 0.95 and 0.84 in the training and test cohorts, respectively (54).

Li et al. developed an integrated model incorporating radiomics, clinical risk factors, and ML for 108 HCC patients who continued TACE after becoming refractory. BCLC stage and lymphocyte-to-monocyte ratio (LMR) were identified as independent prognostic factors. The SVM-based combined model achieved AUCs of 0.956 (95% CI: 0.910–1.000) and 0.897 (95% CI: 0.772–1.000) in the training and test sets, respectively, significantly outperforming models based on clinical or radiomic data alone (46). Jin et al. demonstrated that imaging biomarkers can decode tumor heterogeneity and predict response to TACE combined with immunotherapy and targeted therapy (55). These AI-driven approaches hold considerable promise for facilitating more precise, individualized clinical decision-making throughout the TACE treatment course.

## 6 TACE combination therapy and AI-assisted decision-making

Given the inherent limitations of TACE monotherapy, TACE combined with systemic therapy has emerged as a key strategy for improving efficacy and overcoming refractoriness. The PROLONG trial—a phase III, open-label, multicenter randomized controlled trial—evaluated sorafenib plus TACE (SOR-TACE) versus TACE alone in patients with recurrent intermediate-stage HCC and microvascular invasion (MVI) (56). Among 162 enrolled patients, SOR-TACE yielded a significantly longer median OS than TACE monotherapy (22.2 months *vs*. 15.1 months; HR: 0.55; P < 0.001), along with prolonged progression-free survival (16.2 months *vs*. 11.8 months) (56).

TACE combined with immune-checkpoint inhibitors (e.g., sintilimab and other PD-1 inhibitors) is also under active investigation. Li et al. reported the efficacy of TACE plus sintilimab in intermediate-stage HCC patients exceeding the up-to-seven criteria, using radiomic analysis to identify predictive factors influencing treatment outcomes (57). The role of AI in combination-therapy settings extends beyond response prediction to encompass optimal regimen selection, treatment-timing optimization, and adverse-event monitoring.

**Figure 4:**
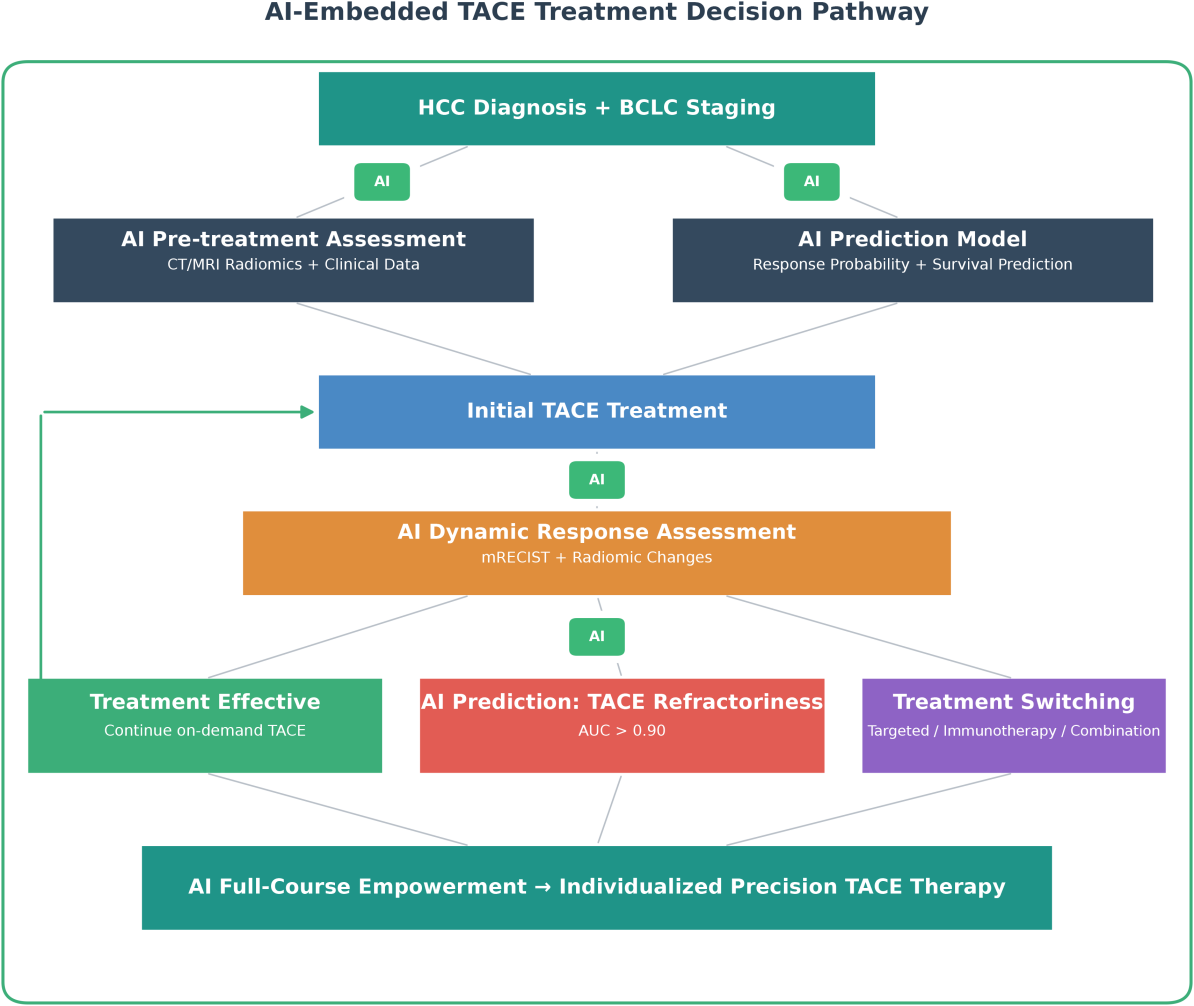
AI-embedded TACE treatment decision pathway. AI provides support at each critical decision node, from pre-treatment assessment and post-procedural dynamic monitoring to refractoriness determination and therapeutic switching, enabling individualized, full-course empowerment of precision TACE therapy.

In the domain of TACE technique selection (cTACE *vs*. DEB-TACE *vs*. DSM-TACE), AI has also shown potential for individualized decision support. Radiomics-based predictive models can stratify patients according to their predicted response probabilities for different TACE modalities, identifying those most likely to benefit from a specific technique and facilitating a transition from experience-driven “one-size-fits-all” approaches to individualized interventions is illustrated in **Figure 4** (58).

## 7 Current challenges and limitations

### 7.1 The generalization gap: magnitude, causes, and mitigation

Despite the encouraging discriminative performance summarized above, several challenges constrain the clinical translation of AI in TACE; these are organized below by theme and summarized, alongside corresponding future directions, in **Table 4**. Across the studies summarized in **Table 1**, a consistent pattern emerges: AUROC declines from training to internal validation by a median of approximately 0.05–0.08, and declines further from internal to external validation by an additional 0.08–0.15. The meta-analysis of Kiani et al. reported pooled AUROCs of 0.89 for internal versus 0.81 for external validation (42), and refractoriness models such as the HRD score of Dai et al. (49) exhibit the steeper end of this gap. This generalization gap is the single largest barrier between current AI evidence and routine clinical deployment, and on the basis of present data, reported AUROCs from single-center retrospective studies should be interpreted as upper bounds of achievable real-world performance.

**Table 4:** Strengths, limitations, and future directions of AI across TACE application scenarios.

Application	Strengths	Limitations	Future directions
Response prediction	AUROC 0.81–0.95; combined models outperform single-source; supports patient selection	Predominantly retrospective; limited external validation; low IBSI compliance	Multicenter prospective validation; standardized feature extraction; real-world implementation
Survival estimation	Predicts 1–3-year OS; DL excels at long-term prediction; multimodal fusion improves accuracy	Wide train–validation gap; small samples; limited generalizability	Large-scale datasets; dynamic prediction models; integration with clinical scoring systems
Refractoriness prediction	AUC 0.90–0.96; enables early identification; avoids unnecessary repeat TACE	Definitional controversy; geographic bias; limited interpretability	Unified refractoriness criteria (CCI *vs*. JSH *vs*. BCLC); integration with molecular mechanisms; XAI for clinical adoption
Technique selection	Predicts response probability by technique; individualized matching; reduces futile procedures	Single-center data; short-term endpoints; no long-term survival validation	Prospective RCT validation; cost-effectiveness analyses; clinical decision-system integration

The causes of the gap are well characterized in the broader radiomics literature but remain only sporadically addressed in TACE-specific studies. They include: (a) scanner and protocol heterogeneity—CT slice thickness, MRI field strength, contrast timing, and reconstruction kernel all materially alter texture features; (b) population shift—most cited cohorts are HBV-predominant Chinese populations, whereas many Western HCC cohorts are HCV- or MASLD-predominant, with different tumor biology and ancillary findings; (c) label-definition shift—mRECIST versus RECIST 1.1, and JSH versus BCLC versus CCI refractoriness criteria, yield meaningfully different outcome distributions; and (d) overfitting under small samples—radiomic feature counts often exceed event counts, and LASSO does not fully prevent leakage when feature selection is performed before nested cross-validation.

Mitigation strategies that have shown promise in adjacent oncologic domains but remain underused in TACE include: ComBat harmonization, which can realign radiomic features computed from heterogeneous CT protocols and scanners without requiring access to the underlying images (59); federated learning, which enables cross-institutional training without raw-data sharing and has achieved near-centralized performance on multi-institutional radiology tasks (60); domain-adaptation techniques for population shift; and transfer learning from large pre-trained vision foundation models. Equally important are study-design fixes—pre-registered analysis plans, independent external test cohorts withheld until final evaluation, and rigorous events-per-variable accounting—which together must become routine before AI models can reliably support TACE decisions in unfamiliar populations.

### 7.2 Limited sample sizes and geographic bias

Existing studies are generally limited by small sample sizes, and the overwhelming majority originate from China, resulting in insufficient geographic representativeness (28). Significant differences in patient demographics, etiological profiles (HBV *vs*. HCV *vs*. MASLD-related), and clinical characteristics across regions may limit the global generalizability of study findings. Furthermore, no published study in this field has reported *a priori* statistical power calculations, raising concerns about underpoweredness and the risk of type II errors (28).

### 7.3 Low standardization

Substantial heterogeneity exists across studies with respect to radiomic feature extraction protocols, ROI segmentation methods, imaging acquisition parameters, and outcome assessment criteria. The absence of uniform IBSI-compliant reporting renders cross-platform and cross-study result comparison and replication difficult (28, 29). Inconsistent criteria for evaluating TACE response (e.g., mRECIST *vs*. RECIST 1.1) further compromise the comparability of published findings.

### 7.4 Interpretability and clinical actionability

DL models are frequently regarded as “black boxes,” with limited decision-process transparency—a significant obstacle to clinical adoption (61). The development of explainable AI (XAI) techniques holds promise for addressing this issue, although XAI applications in the TACE domain remain nascent. Additionally, a consistent absence of cost-effectiveness analyses across existing studies precludes a definitive assessment of the incremental clinical value of radiomics over currently available biomarkers (27).

## 8 Future perspectives

The path from retrospective AI proof-of-concept to clinically deployed TACE decision support requires research directions that are specific to interventional hepatology, rather than generic recommendations applicable to all medical AI. We therefore propose five TACE-anchored priorities that distinguish this field’s next phase from the broader medical-AI research agenda.

1. Intraprocedural AI guidance, not only pre-procedural prediction. Almost all current TACE-AI models operate on pre-procedural CT or MRI. The largest unmet opportunity lies in real-time AI integration with cone-beam CT (CBCT) and digital subtraction angiography (DSA) during the TACE procedure itself: automated tumor-feeding artery detection, lipiodol deposition quantification, and intraprocedural endpoint determination. Multicenter evidence already shows that CBCT with automated tumor-feeder detection software more than doubles the rate of identifying small tumor-feeding branches relative to conventional DSA and independently improves OS in unresectable HCC (HR 0.38, P < 0.001) (62). Deep-learning analysis of intraprocedural DSA videos can additionally provide real-time, automatic prediction of treatment response and a quantitative tumor-segmentation overlay during the procedure (63). Embedding such tools in routine TACE workflows addresses the operator-dependent variability that currently confounds outcomes more than any pre-procedural prediction can.
2. Refractoriness as a dynamic, time-to-event AI prediction target. The ongoing definitional debate around TACE refractoriness (Section 5.1) is in part an artifact of forcing a binary label onto a continuous biological process. AI models that output a time-to-refractoriness hazard, conditional on the response to the first and second TACE sessions and on serial liver-function trajectories, would map more naturally onto the clinical decision of when to switch to systemic therapy than the current cross-sectional classifiers. Such dynamic models would also help reconcile the divergent JSH, BCLC, and CCI criteria by making the underlying continuous risk explicit.
3. Technique-selection models with paired counterfactual outcomes. Whether a given patient benefits more from cTACE, DEB-TACE, or DSM-TACE is a causal question that current observational radiomics models cannot answer. Future studies should adopt causal-inference frameworks (e.g., uplift modeling, double machine learning) on multi-arm cohorts, or design AI-stratified randomized trials, to move technique selection beyond simple response-probability matching (58).
4. Joint modeling of tumor response and hepatic functional reserve. Repeat TACE failures are often driven not by tumor biology but by progressive deterioration of liver function. Integrating AI-derived volumetric liver-function measures—such as gadoxetic acid–enhanced MRI hepatocyte fraction and hepatobiliary-phase parenchymal enhancement metrics, which are increasingly validated as quantitative liver-function biomarkers in HCC populations (64)—into TACE outcome models would allow joint optimization of anti-tumor efficacy and parenchymal preservation, a uniquely locoregional concern that systemic-therapy AI models do not face.
5. Mechanism-anchored AI for next-generation embolic materials. The molecular mechanisms of refractoriness summarized in Section 5.3 point to specific therapeutic vulnerabilities—acidic microenvironment, HIF-1α activation, autophagy, neoangiogenesis—that can in principle be exploited by next-generation embolic materials such as pH-responsive microspheres or HIF-1α–targeting drug-eluting beads (52). AI-driven inverse design, in which desired imaging–pharmacokinetic phenotypes constrain the selection of material properties, could meaningfully accelerate translational development at the intersection of materials science and interventional oncology.

Adoption of harmonized reporting standards (TRIPOD+AI, CLAIM) and rigorous risk-of-bias appraisal (PROBAST, QUADAS-AI) (30–33) remains a necessary cross-cutting requirement for all five directions above, but is no longer a sufficient research agenda in itself.

## 9 Conclusions

AI technologies—particularly radiomics and DL—have demonstrated considerable promise across multiple stages of TACE treatment for HCC, including response prediction, survival estimation, and refractoriness assessment. AI models based on radiomics and/or DL have achieved high discriminative performance (AUROC: 0.81–0.92) for predicting TACE outcomes, with combined clinico-radiological multimodal models consistently outperforming single-source approaches. Among the algorithms evaluated, CNNs and gradient-boosting, support-vector-machine, and random-forest models exhibited the strongest performance. Key predictive features include ALBI grade, BCLC stage, AFP level, tumor diameter, distribution, and peritumoral arterial-phase enhancement.

However, AI research in the TACE field remains constrained by insufficient external validation, geographically biased cohorts dominated by HBV-related disease, limited adherence to IBSI/TRIPOD+AI/CLAIM reporting standards, and a generalization gap of approximately 0.10 AUROC between internal and external validation. Future progress will depend on the five TACE-specific priorities outlined above: intraprocedural CBCT/DSA AI guidance, dynamic time-to-refractoriness modeling, causal technique-selection inference, joint modeling of tumor response with hepatic functional reserve, and mechanism-anchored AI for next-generation embolic materials. Pursuit of these directions, anchored in standardized methodology and external validation, will be instrumental in transitioning AI from a research tool to a clinically actionable decision-support system—ultimately advancing precision, individualized TACE therapy for patients with HCC.
